# Molecular Basis of Surfactin-Induced Macrophage Modulation and Its Implications in Medication-Related Osteonecrosis of the Jaw Pathogenesis

**DOI:** 10.3390/ijms27031157

**Published:** 2026-01-23

**Authors:** Yuki Kodama-Maruyama, Hiroki Tsurushima, Ayaka Koga, Yoshie Nagai-Yoshioka, Ryota Yamasaki, Manabu Habu, Izumi Yoshioka, Wataru Ariyoshi

**Affiliations:** 1 Division of Infections and Molecular Biology, Department of Advanced Pathophysiological Science, Kyushu Dental University, Kitakyushu 803-8580, Fukuoka, Japan; r21kodama@fa.kyu-dent.ac.jp (Y.K.-M.); r20koga@fa.kyu-dent.ac.jp (A.K.); r16yoshioka@fa.kyu-dent.ac.jp (Y.N.-Y.); r18yamasaki@fa.kyu-dent.ac.jp (R.Y.); 2 Division of Oral Medicine, Department of Oral and Maxillofacial Disease Control, Kyushu Dental University, Kitakyushu 803-8580, Fukuoka, Japan; r13yoshioka@fa.kyu-dent.ac.jp; 3 Department of Health Sciences, Kyushu Dental University, Kitakyushu 803-8580, Fukuoka, Japan; 4 Oral Medicine Innovation Center, Kyushu Dental University, Kitakyushu 803-8580, Fukuoka, Japan; 5 Division of Oral and Maxillofacial Surgery, Department of Oral and Maxillofacial Disease Control, Kyushu Dental University, Kitakyushu 803-8580, Fukuoka, Japan; h-manabu@kyu-dent.ac.jp

**Keywords:** surfactin, IL-6, AP-1, MAPK, JAK/STAT, MRONJ

## Abstract

Medication-related osteonecrosis of the jaw (MRONJ) is a refractory disease for which no established treatment currently exists. Surfactin, a biosurfactant produced by *Bacillus subtilis*, exhibits antimicrobial activity, anticancer effects, and anti-inflammatory properties, suggesting its potential medical applications. This study aimed to elucidate the ability of surfactin to modulate the immune response induced by lipopolysaccharide (LPS) derived from periodontal pathogens (*Aggregatibacter actinomycetemcomitans*), clarify the underlying molecular mechanisms, and explore its potential utility in the treatment of MRONJ. Reverse transcription quantitative polymerase chain reaction demonstrated that surfactin suppresses LPS-induced interleukin-6 (IL-6) expression and secretion in J774.1 cells in a concentration-dependent manner. Western blot analysis showed that surfactin inhibited activation of the JNK-c-Jun-AP-1 axis and the JAK/STAT signaling pathways in J774.1 cells. The effects of surfactin administration were further evaluated in an in vivo MRONJ model. Co-treatment with surfactin significantly reduced the extent of LPS-induced bone necrosis. Overall, these findings suggest that surfactin suppresses LPS-induced IL-6 expression in macrophages and inhibits osteonecrosis induced by bisphosphonate preparations and LPS through negative regulation of the JNK-c-Jun-AP-1 axis and inhibition of the JAK/STAT pathway. Hence, surfactin may represent a promising candidate for MRONJ management.

## 1. Introduction

Since the first case of medication-related osteonecrosis of the jaw (MRONJ) was reported by Marx et al. in 2003 [1], the number of patients with this condition has been increasing. MRONJ is a form of osteonecrosis that occurs specifically in the jawbone and is associated with medications such as bisphosphonates. MRONJ is defined by bone exposure or fistula formation in the oral and maxillofacial regions persisting for more than eight weeks. A scoping review of 92 papers indicated that the incidence of MRONJ was 6.22% among patients with tumor and 0.58% among patients with osteoporosis [2]. MRONJ is an extremely refractory disease, and in advanced cases, mandibular resection may be required; hence, the condition affects patients’ quality of life considerably. Despite this, definitive treatment or management strategies for MRONJ are yet to be established. Although the pathogenesis of this disease is not fully understood, various studies have proposed several hypotheses, including the involvement of suppressed bone remodeling and impaired angiogenesis [3,4]. One such hypothesis highlights the role of lipopolysaccharide (LPS), a pathogenic factor produced by Gram-negative bacteria that colonize the oral cavity [5].

Interleukin-6 (IL-6), produced by various cell types including monocytes, fibroblasts, and endothelial cells, is a multifunctional cytokine that regulates immune responses and inflammatory reactions by controlling cell proliferation, differentiation, and survival [6]. IL-6 is involved in the pathogenesis of many diseases, such as chronic inflammation, including rheumatoid arthritis and autoimmune disorders, as well as bacterial infections [7]. Studies using clinical specimens from MRONJ patients have reported an increased proportion of pro-inflammatory M1 macrophages and overexpression of IL-6 in the soft tissues adjacent to necrotic bone in Stage 2 and Stage 3 diseases according to the MRONJ staging classification (AAOMS 2014) [8]. Macrophages are one of the primary IL-6-producing cells, and their expression is induced by stimulatory factors such as interferon-γ, tumor necrosis factor-α (TNF-α), and LPS [9]. The induction of IL-6 expression by these stimulatory factors involves multiple regulatory mechanisms, including activation of intracellular signaling pathways such as nuclear factor-kappa B (NF-κB), mitogen-activated protein kinase (MAPK), CCAAT/enhancer-binding protein beta (C/EBPβ), Janus kinase (JAK)/signal transducers and activator of transcription (STAT), and phosphoinositide 3-kinase (PI3K)/AKT, as well as post-transcriptional modifications [10,11,12,13,14]. Therefore, we considered regulating IL-6 production by macrophages as an essential strategy for controlling the pathogenesis of MRONJ.

Biosurfactants (surfactants produced by microorganisms) have been widely researched and applied in many fields due to their excellent biodegradability and high biocompatibility [15]. Surfactin, a biosurfactant fermented by *Bacillus subtilis*, is used as a surfactant and emulsifier. Although surfactin has not been approved for clinical use, it exhibits antimicrobial [16], anticancer [17], and anti-inflammatory activities [18], supporting its potential applicability in medical settings. However, reports regarding its role in the pathogenesis of MRONJ and its regulatory effects on IL-6 production remain limited.

This study aimed to elucidate the ability of surfactin to modulate the immune response induced by LPS derived from periodontal pathogens, clarify its underlying molecular mechanisms, and explore its potential application in the treatment of MRONJ.

## 2. Results

### 2.1. Effect of Surfactin on Cell Viability of J774.1 Cells

**Figure 1:**
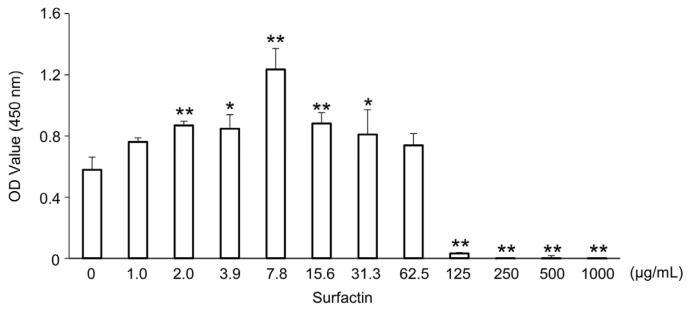
Effects of surfactin on the proliferation of J774.1 cells. J774.1 cells were stimulated with indicated concentrations of surfactin (0–1000 μg/mL) for 48 h. The live cells were detected by the CCK-8 assay. (* *p* < 0.05, ** *p* < 0.01). OD, optical density; CCK-8 assay, cell counting kit-8 assay.

The effect of surfactin on the proliferation of J774.1 cells was evaluated using the WST-8 assay. No significant effect of surfactin on cell viability was observed at concentrations up to 62.5 μg/mL. (Figure 1).

### 2.2. Surfactin Concentration-Dependently Suppresses LPS-Induced IL-6 Expression and Secretion in J774.1 Cells

**Figure 2:**
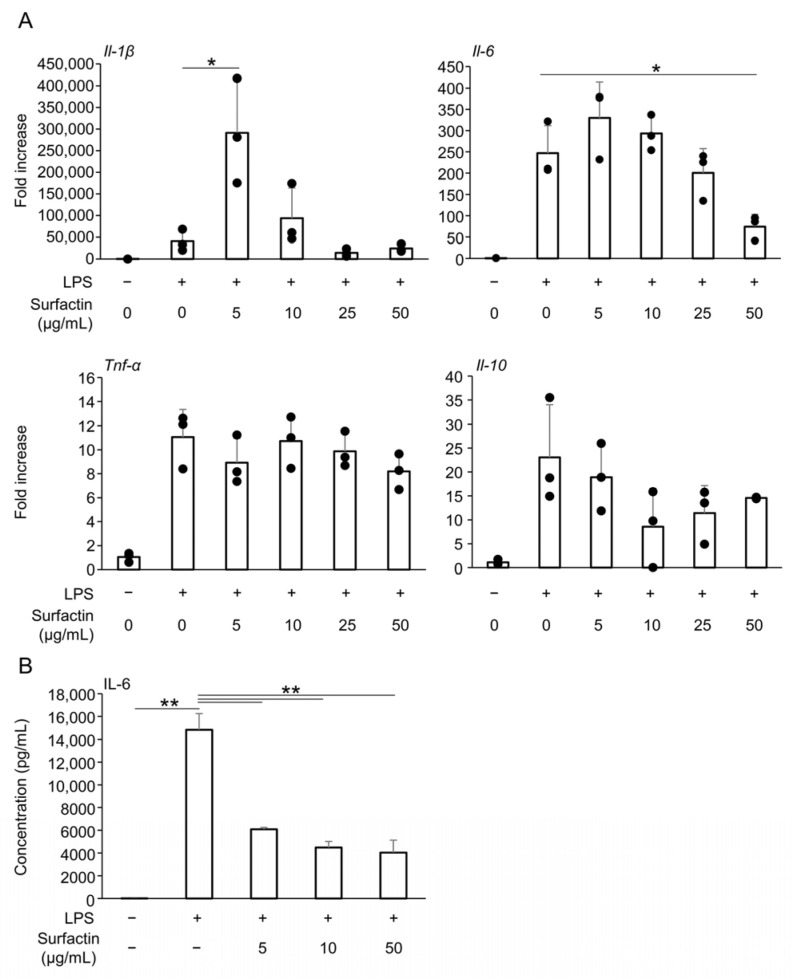
The effect of surfactin on the expression of inflammatory cytokines. (**A**) J774.1 cells were stimulated with surfactin (0, 5, 10, 25, and 50 μg/mL) in the presence of LPS (100 ng/mL) for 2 h. The expressions of *Il-1β*, *Il-6*, *Tnf-α*, and *Il-10* were quantified by RT-qPCR. (**B**) J774.1 cells were stimulated with surfactin (0, 5, 10, and 50 μg/mL) in the presence of LPS (100 ng/mL) for 24 h. IL-6 proteins in the conditioned media were quantified by ELISA. (* *p* < 0.05, ** *p* < 0.01). LPS, lipopolysaccharide; RT-qPCR, quantitative reverse transcription polymerase chain reaction; IL, interleukin; TNF-α, tumor necrosis factor α; ELISA, enzyme-linked immunosorbent assay.

Reverse transcription quantitative polymerase chain reaction (RT-qPCR) was performed to examine the effect of surfactin on major pro-inflammatory and anti-inflammatory cytokines induced by LPS. Treatment with surfactin at concentrations of 50 μg/mL significantly suppressed LPS-induced *Il-6* mRNA expression in a concentration-dependent manner. However, no significant inhibitory effect of surfactin was observed on the induction of inflammatory cytokines *Il-1β* and *Tnf-α*, or the anti-inflammatory cytokine *Il-10*, by LPS (Figure 2A). Additionally, enzyme-linked immunosorbent assay (ELISA) showed that surfactin significantly suppressed LPS-induced secretion of IL-6 protein in J774.1 cells (Figure 2B).

### 2.3. Surfactin Suppresses LPS-Induced Il-6 Expression in RAW264.7 Cells

**Figure 3:**
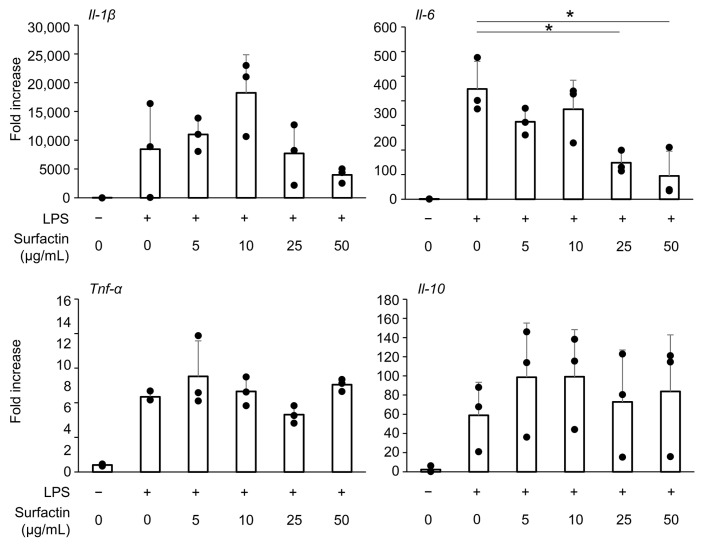
The effect of surfactin on gene expression of inflammatory cytokines in RAW264.7 cells. RAW 264.7 cells were stimulated with surfactin (0, 5, 10, 25, and 50 μg/mL) in the presence of LPS (100 ng/mL) for 2 h. The expressions of *Il-1β*, *Il-6*, *Tnf-α*, and *Il-10* were quantified by RT-qPCR. (* *p* < 0.05).

The effect of surfactin on the induction of inflammatory cytokine expression in another mouse macrophage-like cell line, RAW264.7 cells, was evaluated. We have already found that administration of surfactant at concentrations of 50 μg/mL or less does not inhibit the proliferation of RAW264.7 cells by CCK-8 assay [19]. Similarly to observations in J774.1 cells, surfactin suppressed LPS-induced *Il-6* mRNA expression, while not affecting the expression of *Il-1β*, *Tnf-α*, or *Il-10* (Figure 3).

### 2.4. Surfactin Has No Effect on the Expression of Negative Regulators of IL-6

**Figure 4:**
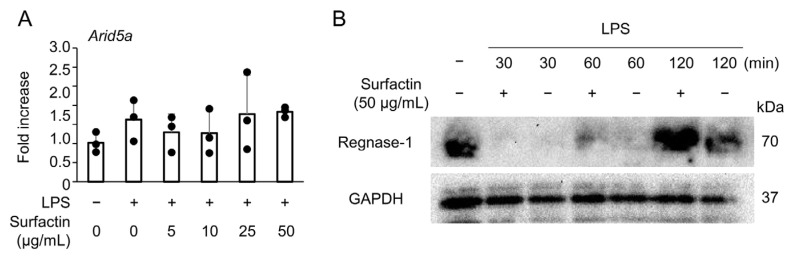
The effect of surfactin on negative regulators of IL-6. (**A**) J774.1 cells were stimulated with surfactin (0, 5, 10, 25, and 50 μg/mL) in the presence of LPS (100 ng/mL) for 2 h. The expression of *Arid5a* was quantified by RT-qPCR. (**B**) J774.1 cells were stimulated with LPS (100 ng/mL) and surfactin (50 μg/mL) for 30, 60, or 120 min. Protein levels of Regnase-1 were detected by Western blot analysis. GAPDH served as a loading control.

To elucidate the molecular mechanism underlying the suppression of LPS-induced IL-6 expression by surfactin, we focused on the expression of AT-rich interactive domain-containing protein 5a (Arid5a) and Regulatory RNase 1 (Regnase-1), negative regulators of IL-6. No significant changes in *Arid5a* mRNA levels were observed following the addition of either LPS or surfactin (Figure 4A). Conversely, Regnase-1 protein was induced by the 120-min stimulation of LPS, and its expression was further enhanced by co-treatment with surfactin (Figure 4B).

### 2.5. Surfactin Suppresses LPS-Induced IL-6 Expression via Down-Regulation of JNK-Mediated c-Jun Activation

**Figure 5:**
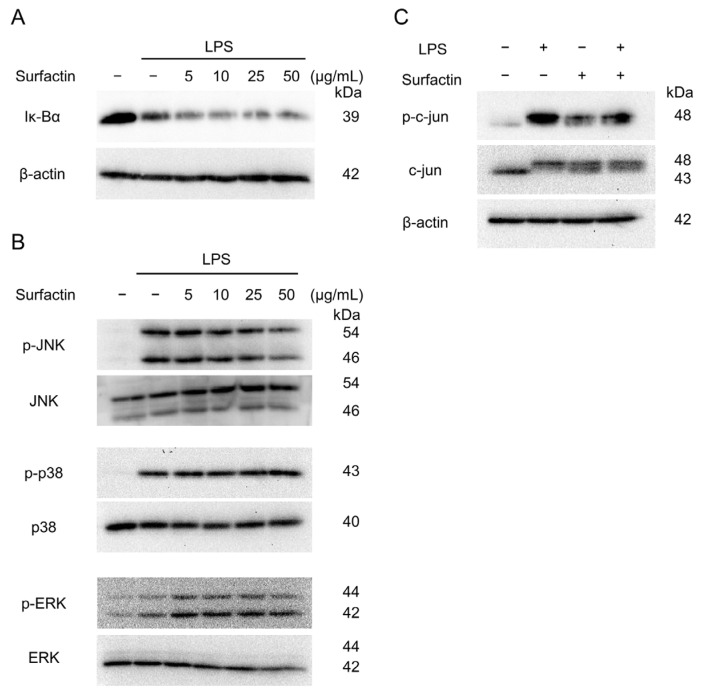
The effect of surfactin on NF-κB and MAPK-mediated signaling pathway activated by LPS. J774.1 cells were stimulated with LPS (100 ng/mL) and surfactin (0, 5, 10, 25, and 50 μg/mL) for 15 min (**A**) or 30 min (**B**). Protein expressions of IκBα (**A**), phosphorylated JNK (p-JNK), JNK, phosphorylated ERK (p-ERK), ERK, phosphorylated p38 MAPK (p-p38), and p38 MAPK (p38) were detected by Western blot analysis. β-actin served as a loading control. (**C**) J774.1 cells were stimulated with LPS (100 ng/mL) and surfactin (50 μg/mL) for 30 min. Protein expression of phosphorylated c-Jun (p-c-Jun) and c-Jun was detected by Western blot analysis. β-actin served as a loading control. MAPK, mitogen-activated protein kinase.

To elucidate the intracellular signaling involved in the suppression of LPS-induced IL-6 expression by surfactin, the activation of the NF-κB and MAPK pathway was examined by Western blotting. Surfactin had no effect on LPS-induced degradation of IκBα protein (Figure 5A). Regarding MAPK component proteins, surfactin inhibited LPS-induced JNK phosphorylation in a concentration-dependent manner, but did not affect the phosphorylation levels of p38 and ERK protein (Figure 5B). Furthermore, surfactin inhibited the phosphorylation of c-Jun, a component of AP-1 downstream of the JNK-mediated signaling pathway (Figure 5C).

**Figure 6:**
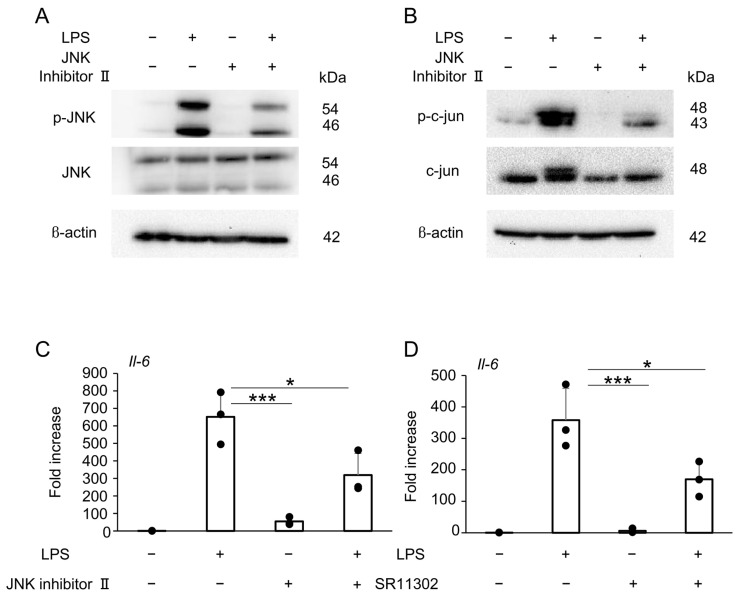
The effect of surfactin on the JNK-AP-1-mediated signaling pathway activated by LPS. (**A**) J774.1 cells were pretreated with JNK Inhibitor II (50 μM) for 1 h and stimulated with LPS (100 ng/mL) for 30 min. Each protein expression level was detected by Western blot analysis. β-actin served as a loading control. (**B**) J774.1 cells were pretreated with JNK Inhibitor II (50 μM) for 1 h and stimulated with LPS (100 ng/mL) for 30 min. Each protein expression level was detected by Western blot analysis. β-actin served as a loading control. (**C**) J774.1 cells were pretreated with JNK Inhibitor II (50 μM) for 1 h and stimulated with LPS (100 ng/mL) for 2 h. The mRNA expression of *Il-6* was detected by RT-qPCR analysis. (**D**) J774.1 cells were pretreated with SR11302 (50 μM) for 30 min and stimulated with LPS (100 ng/mL) for 2 h. The mRNA expression of *Il-6* was detected by RT-qPCR analysis. (* *p* < 0.05, *** *p* < 0.001).

Pretreatment with JNK inhibitor II effectively suppressed LPS-induced phosphorylation of JNK (Figure 6A) and c-Jun (Figure 6B). Moreover, RT-qPCR revealed that LPS-induced *Il-6* expression was significantly suppressed by pretreatment with either JNK inhibitor II (Figure 6C) or SR11302, an inhibitor of AP-1 transcriptional activity (Figure 6D).

### 2.6. Surfactin Suppresses LPS-Induced IL-6 Expression via Down-Regulation of JAK/STAT Signaling Pathway

**Figure 7:**
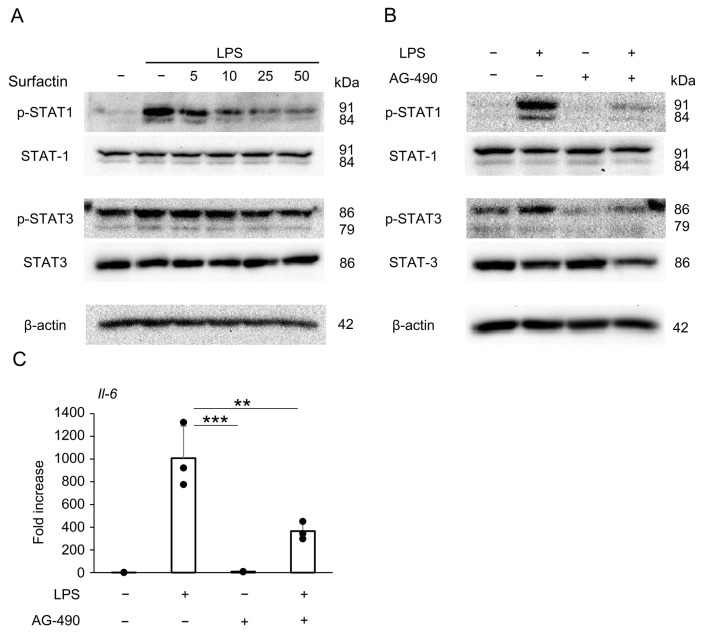
The effect of surfactin on the JAK-STAT-mediated signaling pathway activated by LPS. (**A**) J774.1 cells were stimulated with LPS (100 ng/mL) and surfactin (0, 5, 10, 25, and 50 μg/mL) for 2 h. (**B**) J774.1 cells were pretreated with AG-490 (30 μM) for 30 min and stimulated with LPS (100 ng/mL) for 2 h. Each protein expression level was detected by Western blot analysis. β-actin served as a loading control. (**C**) J774.1 cells were pretreated with AG-490 (30 μM) for 30 min and stimulated with LPS (100 ng/mL) for 2 h. The mRNA expression of *Il-6* was detected by RT-qPCR analysis. (** *p* < 0.01, *** *p* < 0.001).

To identify signaling pathways other than the JNK/c-Jun axis involved in the downregulation of IL-6 expression by surfactin, we focused on the JAK/STAT pathway and revealed that surfactin suppressed LPS-induced STAT1 phosphorylation (Figure 7A). Pretreatment with the JAK2 inhibitor AG-490 downregulated both LPS-induced STAT1 and STAT3 phosphorylation (Figure 7B). Furthermore, AG-490 pretreatment was shown to suppress *Il-6* mRNA expression (Figure 7C).

### 2.7. Surfactin Suppresses Bone Necrosis in MRONJ Animal Models

**Figure 8:**
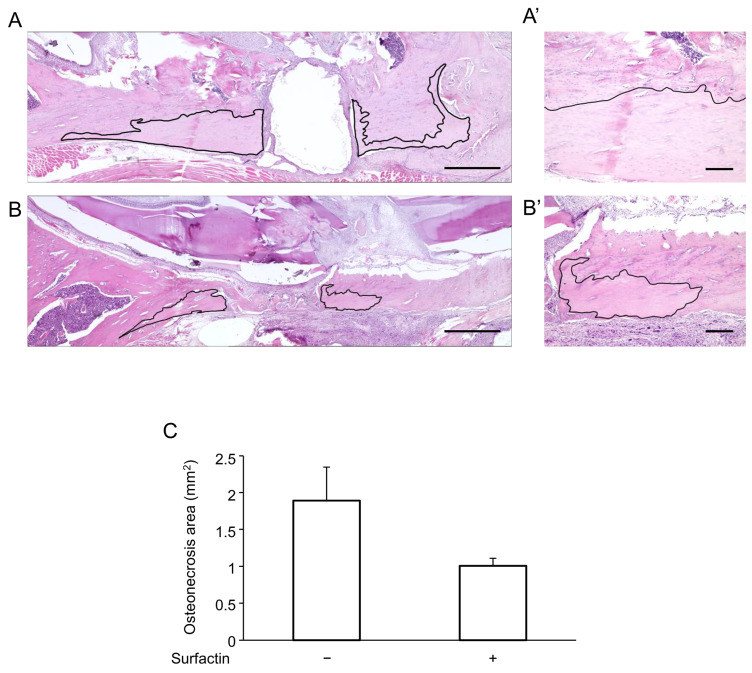
Effects of surfactin on osteonecrosis. Comparison of mandibular osteonecrosis extent by H-E staining. (**A**) Representative image of the LPS-only group. (**A’**) High-magnification view of (**A**). (**B**) Representative image of the LPS and surfactin co-treatment group. (**B’**) High-magnification view of (**B**). The area outlined in black represents the necrosis area. Scale bars: (**A**,**B**) 1 mm, (**A’**,**B’**) 200 μm. (**C**) Comparison of the area of osteonecrosis. H-E, hematoxylin and eosin.

Finally, the effects of surfactin administration on MRONJ animal models were verified in vivo. Following LPS packing into the mandibular defect, bone necrosis lesions accompanied by the disappearance of osteocyte nuclei were observed in the surrounding area (Figure 8A). Co-administration of surfactin showed a tendency to reduce the extent of LPS-induced bone necrosis (Figure 8B,C).

## 3. Discussion

MRONJ is a multifactorial disease. Although its pathophysiological mechanism is not fully understood, it is thought to involve multiple factors such as impaired bone remodeling [20] and inhibition of angiogenesis [21,22]. Furthermore, the involvement of inflammation and bacterial infection has been suggested [20], and in vivo studies have demonstrated that LPS derived from periodontal pathogens can induce MRONJ [23].

LPS, a component of the cell wall of Gram-negative bacteria, is known to act on macrophages to induce inflammatory responses [9]. J774.1 and RAW264.7 mouse monocyte/macrophage cell lines were used in this study, as both are known to respond to LPS from periodontal bacteria. Consistent with previous reports [24,25], 100 ng/mL of LPS derived from *Aggregatibacter actinomycetemcomitans* enhanced the gene expression of various inflammatory and anti-inflammatory cytokines (Figure 2A). Furthermore, we found that LPS concentrations below 400 ng/mL did not significantly inhibit the proliferation of J774.1 cells (Supplemental Figure S1). In this induction pathway, we found that adding surfactin at non-cytotoxic concentrations negatively regulated IL-1β and IL-6 expression, suggesting that surfactin exerts specific inhibitory activity on these cytokines. Therefore, this study focused on surfactin’s ability to regulate IL-6 expression and conducted subsequent investigations. IL-6 is rapidly secreted after signal peptide removal in the rough endoplasmic reticulum immediately following synthesis, followed by processing and maturation. Surfactin was also confirmed to suppress LPS-induced IL-6 secretion (Figure 2B).

Various cell lines have been used in experiments to elucidate macrophage differentiation and function [26]. Similarly to J774.1 cells, the mouse macrophage cell line RAW264.7 cells also demonstrated that surfactin suppresses IL-6 expression, suggesting that surfactin possesses similar modifying effects on LPS-induced inflammatory cytokine production in both macrophage cell lines (Figure 3).

Next, we focused on Regnase-1 (also known as Zc3h12a or MCPIP-1) and Arid5a as post-transcriptional regulatory factors involved in the molecular mechanism of surfactin’s negative control of IL-6. Regnase-1, a nuclease, is known to interact with the stem–loop region present in the 3′-UTR of *Il-6* mRNA, causing its destabilization [27,28]. Conversely, Arid5a inhibits the degradation action of Regnase-1 and acts as a stabilizing molecule for *Il-6* mRNA [29]. Previous reports indicated that Flavipin, an aryl hydrocarbon receptor agonist, regulates IL-6 production via Arid5a modulation [30]. We hypothesized that a similar mechanism might involve surfactin. However, no modification of Regnase-1 or Arid5a expression was observed upon surfactin addition, suggesting that regulation of *Il-6* mRNA stability is not involved in the molecular mechanism underlying the suppression of IL-6 expression (Figure 4).

LPS is recognized by Toll-like receptor 4 (TLR4), a pattern recognition receptor expressed on the cell surface. Subsequently, the adapter protein MyD88 interacts with TLR via the TIR domain, activating various intracellular signals and inducing inflammatory cytokines [31]. As many studies have demonstrated that intracellular signaling molecules such as NF-κB and MAPK are involved in IL-6 expression [32,33], we examined these pathways. Among the MAPK components JNK, ERK, and p38, surfactin was demonstrated to selectively inhibit JNK activation (Figure 5A,B). JNK phosphorylation, induced by diverse extracellular stimuli such as genotoxic stress (e.g., UV irradiation), pro-inflammatory cytokines, and hormones, leads to the activation of c-Jun [34]. Activated c-Jun dimerizes with c-Fos to form the AP-1 complex, which acts as a transcriptional regulator that activates or suppresses target gene transcription [35]. The promoter region of the mouse *Il-6* gene contains an AP-1 binding site that is essential for *Il-6* induction. Surfactin treatment also reduced the phosphorylation levels of both JNK and c-Jun (Figure 5C). Interestingly, treatment with surfactin alone induced a modest increase in c-Jun phosphorylation. A previous study has shown that surfactin from *Bacillus amyloliquefaciens* modulates innate immune responses through activation of signaling pathways such as MAPK, and promotes inflammasome activation [36]. The biological significance of surfactin-mediated MAPK activation in macrophages requires further investigation.

JNK inhibitor II competitively binds to the ATP-binding domain, selectively inhibiting JNK kinase activity. Additionally, SR11302, a retinoid compound, reduces the DNA-binding ability of AP-1 and suppresses its transcriptional activity on the TRE-responsive element it binds to. Pre-treatment with selective inhibitors of JNK and AP-1 significantly suppressed LPS-induced IL-6 expression (Figure 6C,D). These results suggest that surfactin suppresses IL-6 expression by negatively regulating the JNK-c-Jun-AP-1 axis.

Furthermore, the JAK/STAT pathway is one of the intracellular signaling pathways involved in IL-6 expression [37]. In mouse macrophages, it has been reported that binding of LPS to TLR4 directly induces STAT1 phosphorylation [38]. In this study, we found that LPS stimulation derived from periodontal pathogens induced STAT1 protein phosphorylation in J774.1 cells, and this was reversed by co-treatment with surfactin (Figure 7A). STAT proteins activate the JAK family (JAK1, JAK2, JAK3, Tyk2), which induces phosphorylation of tyrosine residues. Then, they form heterodimers or homodimers, translocate to the nucleus, and induce transcription of target genes [39]. Pre-treatment with AG-490, a tyrosine kinase inhibitor that selectively inhibits JAK2, significantly suppressed *Il-6* mRNA expression (Figure 7C) in addition to the phosphorylation of STAT1 and STAT3 (Figure 7B). These results suggest that suppression of the JAK/STAT activation pathway, in addition to the JNK-c-Jun-AP-1 axis, may be involved in the negative regulation of LPS-induced IL-6 production by surfactin. However, there is also a negative feedback mechanism in the inflammatory response where SOCS3, induced by the JAK/STAT pathway, suppresses JAK [40] and TLR4 signaling [41]. Therefore, the ability of surfactin to modify this mechanism also needs to be investigated in the future.

Research using clinical specimens has reported that IL-6 is overexpressed in advanced cases of MRONJ [8]. Furthermore, it has been reported that IL-6 deficiency significantly reduces the incidence of osteonecrosis in mice [42], suggesting IL-6 involvement in MRONJ pathogenesis. Therefore, based on the hypothesis that negative regulation of IL-6 by surfactin regulates MRONJ pathogenesis, we conducted studies using a rat model. Local administration of surfactin was associated with a tendency toward reduced LPS-induced mandibular osteonecrosis (Figure 8), indicating a potential role in suppressing MRONJ pathogenesis. Detailed investigation, including the expression of IL-6 at the local site of mandibular osteonecrosis, is required to clarify the involvement of IL-6 suppression by surfactin in controlling MRONJ pathogenesis. Furthermore, the limitations of this study include the limited number (*n* = 3) of in vivo investigations and the fact that the effects of surfactin on MRONJ pathology were evaluated at only a single time point (4 weeks post-surgery). Further studies with larger sample sizes and longer-term pathological evaluations will be required.

In conclusion, this study demonstrated that surfactin suppresses LPS-induced IL-6 expression in macrophages and inhibits osteonecrosis induced by bisphosphonate preparations and LPS through negative regulation of the JNK-c-Jun-AP-1 axis and inhibition of the JAK/STAT pathway. While stability and solubility evaluation for local lesion administration, along with verification of pharmacological effects and side effects for systemic administration, are necessary, surfactin is expected to be a candidate for MRONJ treatment strategies.

## 4. Materials and Methods

### 4.1. Reagents

LPS was derived from *A. actinomycetemcomitans* using a previously described method [43]. Surfactin was supplied by the Kaneka Corporation (Tokyo, Japan). JNK inhibitor II and Anti-Regnase-1/Zc3h12a (15D11) monoclonal antibody were purchased from Merck Millipore (Darmstadt, Germany). SR11302 was purchased from R&D Systems (Minneapolis, MN, USA). AG-490 was purchased from Cayman Chemical Company (Ann Arbor, MI, USA). Polyclonal antibodies for SAPK/JNK, phospho-SAPK/JNK (Thr183/Tyr185), p38 MAPK, phospho-p38 MAPK (Thr180/Tyr182), STAT1, and phospho-STAT1 (Tyr701) were purchased from Cell Signaling Technology (Beverly, MA, USA). Monoclonal antibodies for p44/42 MAPK (Erk1/2; 137F5), phospho-p44/42 MAPK (Erk1/2; Thr202/Tyr204) (D13.14.4E), IκBα (L35A5), c-Jun (60A8), phospho-c-Jun (Ser63; 54B3), STAT3 (79D7) and phospho-STAT3 (Tyr705; D3A7) were also obtained from Cell Signaling Technology. Anti-β-actin polyclonal antibody was purchased from Sigma Aldrich (St. Louis, MO, USA). GAPDH (6C5) was purchased from Santa Cruz Biotechnology, Inc. (Dallas, TX, USA).

### 4.2. Cell Culture

J774.1 (RCB0434) and RAW 264.7 (RCB0535) cells, mouse macrophage-like cell lineages, were purchased from RIKEN CELL BANK (Ibaraki, Japan). These cells are cultured in RPMI 1640 (FUJIFILM Wako Pure Chemical Corporation, Osaka, Japan) with 10% fetal bovine serum (FBS; Sigma-Aldrich Co., LLC, St. Louis, MO, USA) and 1% penicillin–streptomycin solution (FUJIFILM Wako Pure Chemical Corporation). The cells were incubated at 37 °C in 5% CO_2_. In some experiments, the cells were pretreated with the inhibitors for the indicated times.

### 4.3. WST-8 Assay

J774.1 cells were seeded at 1.0 × 10^4^ cells/well in a 96-well plate and cultured in RPMI 1640 with surfactin for 48 h at 37 °C in 5% CO_2_.

Cell counting kit-8 (CCK-8) kit (DOJINDO Molecular Technologies, Inc., Kumamoto, Japan) was used for the detection of cell proliferation. The cells containing CCK-8 (10 μL/well) were incubated for 2 h. The absorbance was measured at a wavelength of 450 nm using a microplate reader (Multiskan FC; Thermo Fisher Scientific, Rockford, IL, USA).

### 4.4. RT-qPCR

J774.1 and RAW264.7 cells were seeded at 1.0 × 10^6^ cells/well in a 6-well plate and cultured with RPMI 1640 overnight, followed by stimulation with surfactin in the presence of LPS. Total RNA purification and detection of mRNA expression were carried out according to the previous study using primers listed in Table 1 [24].

### 4.5. ELISA

J774.1 cells were seeded at 1.0 × 10^6^ cells/well in a 6-well plate and incubated at 37 °C in 5% CO_2_ overnight, followed by stimulation with surfactin in the presence of LPS for 24 h. The conditioned medium was clarified by centrifugation at 1200 rpm for 5 min.

The mouse IL-6 protein in conditioned media was measured using the ELISA Kit (Quantikine M, 2nd Generation; R&D Systems) according to the manufacturer’s protocol. Absorbance at wavelengths of 450 nm and 540 nm was measured in a microplate reader (Multiskan FC).

### 4.6. Western Blotting

J774.1 cells were seeded at 1.0 × 10^6^ cells/well in a 6-well plate and incubated at 37 °C in 5% CO_2_ overnight, followed by stimulation with surfactin in the presence of LPS. Protein purification from cultured cells and Western blotting were performed as reported previously [24]. Horseradish peroxidase-linked anti-rabbit IgG (Cytiva, Marlborough, MA, USA), anti-mouse IgG (Cytiva), and anti-rat IgG (Santa Cruz, Dallas, TX, USA) were used as secondary antibodies.

### 4.7. Animal Experiment

This animal experiment protocol was approved by the Kyushu Dental University Animal Experiment Committee (No. 23-023). Six 8-week-old male Wistar rats were housed under controlled conditions (temperature 22 ± 1 °C, humidity 50 ± 5%, 12-h light–dark cycle). All rats had free access to water and standard rodent chow. All rats received weekly subcutaneous injections of zoledronic acid monohydrate (FUJIFILM Wako, Tokyo, Japan) at a dose of 0.1 mg/kg body weight for 4 weeks. The zoledronic acid dosage was determined based on the dose administered to adult cancer patients.

Rats were divided into two groups: one receiving LPS alone and another receiving LPS plus surfactin. In the LPS group (*n* = 3), 50 μg of LPS was packed into holes created surgically in both mandibles. In the LPS + surfactin group (*n* = 3), a mixture of 50 μg LPS and 2.5 mg surfactin was packed into the holes. Preliminary experiments identified 50 μg of LPS was sufficient to induce bone necrosis (Supplemental Figure S2). The surfactin dose was determined based on its ability to suppress LPS-induced IL-6 expression in vitro.

Surgery was performed using the method established by Tsurushima et al. [5]. Briefly, a skin incision was made along the rat’s mandibular border. Following blunt dissection, the periosteum of the mandible was incised and retracted to expose the bone surface. Subsequently, under irrigation, the cortical bone was perforated at the posterior border of both mandibles using a 1.6 mm diameter round bur. In each group, LPS or LPS + surfactin dissolved in 10 μL of physiological saline was impregnated into Avitene (Zeria Pharmaceutical, Tokyo, Japan) and packed into the bilateral holes. Finally, the holes were covered and sealed with bone wax (Alfresa-pharma, Osaka, Japan). After thorough irrigation of the wound site, the incision was sutured. Four weeks after surgery, all rats were euthanized, and the mandibles were removed.

The collected mandibles were fixed in 10% formalin, degreased using a mixture of chloroform and ethanol, and decalcified in Decalcifying Solution B (EDTA [pH 7.5]; FUJIFILM Wako) for 10 days at room temperature, and finally paraffin-embedded. Mandible specimens were sectioned sagittally to create 4-μm-thick sections containing the central portion of the perforation site at the mandibular border. The sections were stained with hematoxylin and eosin. Areas of bone necrosis were defined according to Allen and Burr’s criteria as regions where areas of vacuolated osteocyte nuclei exceeded 500 μm^2^ [44]. The area of necrotic bone was measured using an inverted fluorescence phase-contrast microscope (BZ-X810; KEYENCE, Osaka, Japan).

### 4.8. Statistical Analysis

Statistical analysis was performed using EZR software version 1.54. For comparisons involving three or more groups, one-way analysis of variance was used, followed by post hoc tests using Tukey’s or Dunnett’s test. The statistical significance level was set at *p* < 0.05.

## Figures and Tables

**Table 1:** RT-qPCR primer sequences.

Gene	Primer Sequence (5′-3′)
*β-actin*	
Forward	5′-AAG TGT GAC GTT GAC ATC CG-3′
Reverse	5′-TCT GCA TCC TGT CAG CAA TG-3′
*I* *l* *-1β*	
Forward	5′-AAG GGC TGC TTC CAA ACC TTT GAC-3′
Reverse	5′-ATT GCT TGG GAT CCA CAC TCT CCA-3′
*I* *l* *-6*	
Forward	5′-GAG GAT ACC ACT CCC AAC AGA CC-3′
Reverse	5′-ATT GCT TGG GAT CCA CAC TCT CCA ACC TTT GAC-3′
*T* *nf* *-α*	
Forward	5′-TCA TGC ACC ACC ATC AAG GA-3′
Reverse	5′-GAC ATT CGA GGC TCC AGT GAA-3′
*I* *l* *-10*	
Forward	5′-AGG CGC TGT CAT CGA TTT CT-3′
Reverse	5′-TGG AGT CCA GCA GAC TCA AT-3′
*Arid5a*	
Forward	5′-CTG TCC TAC GCA ACA GAC TGG-3′
Reverse	5′-GAA GTG AGG TGC CGC ATA GG-3′

## Data Availability

The original contributions presented in this study are included in the article/Supplementary Material. Further inquiries can be directed to the corresponding authors.

## Supplementary Materials

The following supporting information can be downloaded at: https://www.mdpi.com/article/10.3390/ijms27031157/s1.

## Abbreviations

The following abbreviations are used in this manuscript:

MRONJ	Medication-related osteonecrosis of the jaw
LPS	Lipopolysaccharide
IL-6	Interleukin-6
TNF-α	Tumor necrosis factor-α
NF-κB	nuclear factor-kappa B
MAPK	Mitogen-activated protein kinase
JAK	Janus kinase
STAT	signal transducers and activator of transcription
RT-qPCR	Reverse transcription quantitative polymerase chain reaction
ELISA	Enzyme-linked immunosorbent assay
Arid5a	AT-rich interactive domain-containing protein 5a
Regnase-1	Regulatory RNase 1
TLR4	Toll-like receptor 4
