# Supplementary material for: Molecular Basis of Surfactin-Induced Macrophage Modulation and Its Implications in Medication-Related Osteonecrosis of the Jaw Pathogenesis
Source: Int J Mol Sci. 2026 Jan 23;27(3):1157. doi: 10.3390/ijms27031157 (PMC12897720; doi:10.3390/ijms27031157)
Supplement: Supplementary file 1 [file ijms-27-01157-s001.zip › ijms-4075438-supplementary.pdf]

Supplemental Figure S1

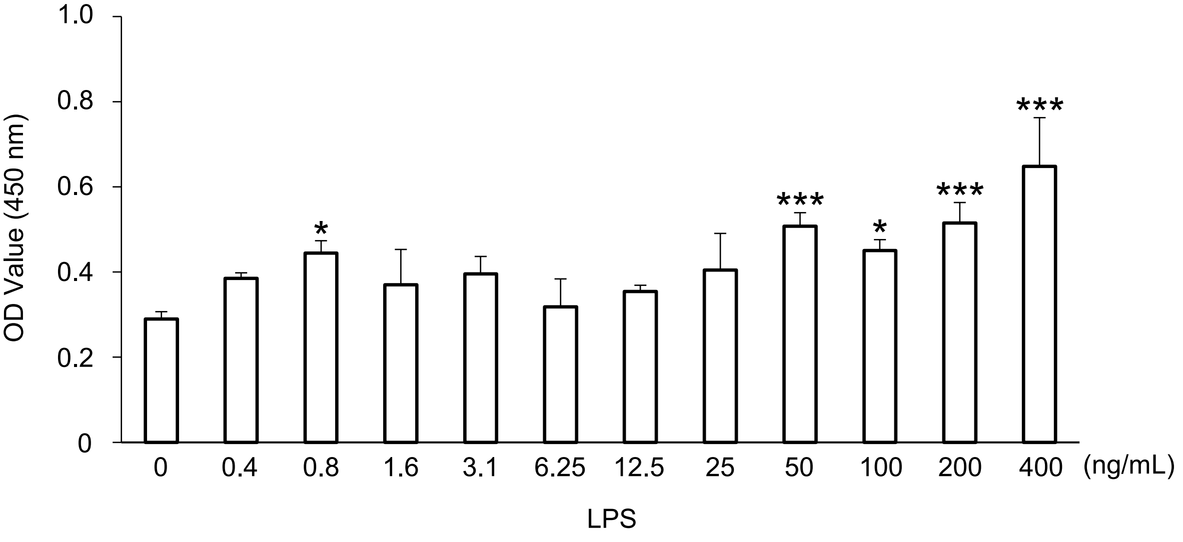

Supplemental Figure S1 Effects of LPS on proliferation of J774.1 cells. J774.1 cells were stimulated with indicated concentrations of LPS (0~400 ng/mL) for 48 h. The live cells were detected by CCK-8 assay. (* $p < 0.05$, ** $p < 0.01$)

Supplemental Figure S2

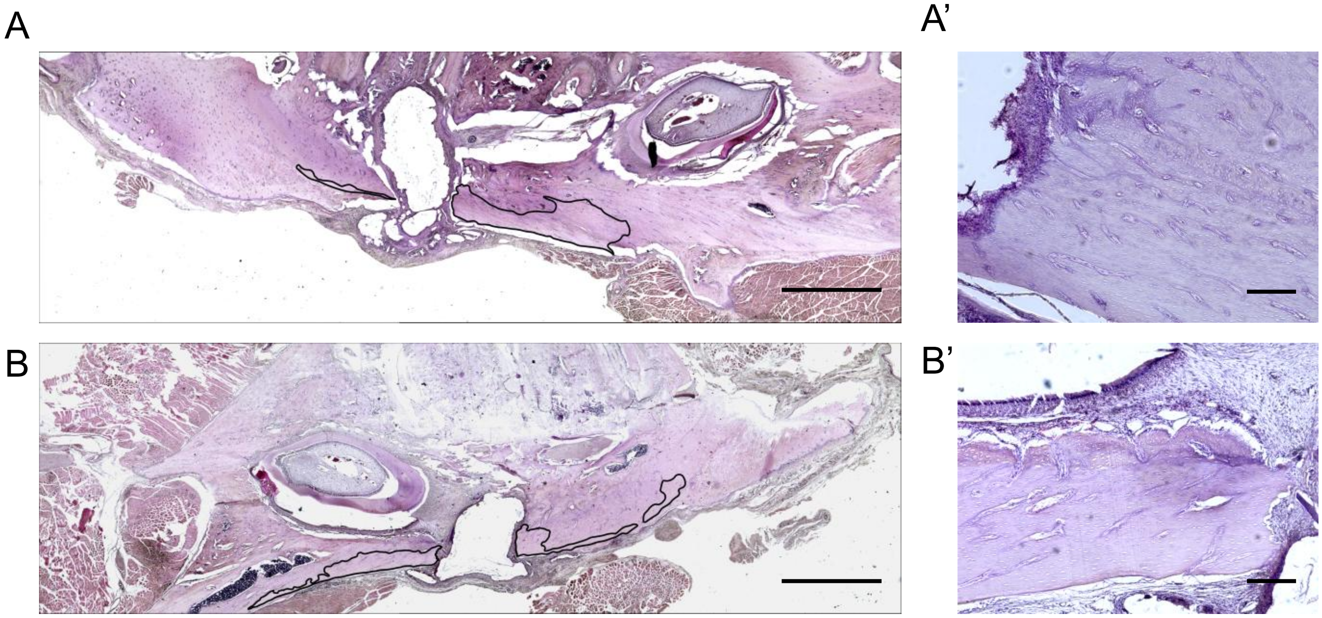

Supplemental Figure S2 Effects of LPS on Osteonecrosis. Comparison of mandibular osteonecrosis extent by H-E staining. (A) Representative image of the LPS 50 µg group. (A’) High-magnification view of (A). (B) Representative image of the LPS 100 µg group. (B’) High-magnification view of (B). The area outlined in black represents the necrosis area. Scale bars: (A) (B) 1 mm, (A’) (B’) 200 µm.
